# High-risk adolescents admitted to acute inpatient psychiatric care: a retrospective clinical analysis of severe externalizing psychopathology and readmission

**DOI:** 10.3389/fpsyt.2026.1756326

**Published:** 2026-02-10

**Authors:** Gyula Sófi

**Affiliations:** Kertváros Pszichológiai Rendelő, Budapest, Hungary

**Keywords:** acute inpatient psychiatry, adolescent hospitalization, conduct disorder, externalizing psychopathology, high-risk adolescents, psychiatric comorbidity, psychiatric readmission, substance use disorders

## Abstract

**Background:**

Adolescents requiring acute inpatient psychiatric care for severe behavioral and emotional dysregulation represent one of the most clinically vulnerable populations within child and adolescent mental health services. While international studies have documented high rates of psychiatric morbidity among adolescents presenting with externalizing pathology and crisis-related admissions, region-specific data from Central and Eastern Europe remain limited. This study aimed to characterize the clinical mental health profile of high-risk adolescents admitted to acute inpatient psychiatric care and to examine patterns of hospitalization and readmission within this population.

**Methods:**

A retrospective cohort study was conducted at a national child and adolescent psychiatric inpatient unit in Hungary. Medical records of adolescents aged 10–19 years admitted between 2009 and 2019 under acute, medically indicated inpatient conditions, including cases involving police or judicial contact, were analyzed. Psychiatric diagnoses were established using standardized assessment tools, including the Mini International Neuropsychiatric Interview for Children and Adolescents (MINI-Kid), Child Behavior Checklist (CBCL), Strengths and Difficulties Questionnaire (SDQ), and Piéron Attention Test. Statistical analyses examined diagnostic prevalence, demographic characteristics, and readmission patterns, including Kaplan–Meier survival analysis of time to readmission.

**Results:**

The final cohort consisted of 570 high-risk adolescents, predominantly male (72.1%), with a mean age of 15.3 years (SD ± 2.1). Psychiatric morbidity was substantial, with conduct disorder (76.2%) and substance use disorders (78.4%) representing the most prevalent diagnoses. Attention-deficit/hyperactivity disorder (17.6%), oppositional defiant disorder (14.9%), post-traumatic stress disorder (10.1%), and psychotic disorders (3.2%) were also identified. One-quarter of participants experienced multiple hospital admissions. While gender was not significantly associated with readmission intervals, increasing age emerged as a significant predictor of earlier readmission, indicating heightened clinical instability among older adolescents.

**Conclusions:**

High-risk adolescents admitted to acute inpatient psychiatric care exhibit an exceptionally high burden of psychiatric disorders, dominated by severe externalizing and substance-related conditions. The findings suggest that acute hospitalization in this population reflects cumulative developmental and psychosocial vulnerability rather than isolated behavioral crises. These results underscore the need for integrated, trauma-informed, and developmentally sensitive psychiatric care pathways, with particular emphasis on early identification, continuity of care, and relapse prevention in clinically unstable adolescents.

## Introduction

Adolescents involved in forensic procedures or admitted under forensic-related circumstances within the healthcare system constitute one of the most psychiatrically vulnerable and clinically complex subgroups within child and adolescent mental health services. A substantial body of international research has demonstrated that justice-involved youth exhibit markedly higher rates of psychiatric disorders compared to community samples, with particularly elevated prevalence of externalizing disorders, substance use disorders, and trauma-related conditions (1–4). These mental health difficulties are closely associated with impaired psychosocial functioning, disrupted developmental trajectories, and an increased risk of repeated system involvement, including recidivism and rehospitalization (5, 6).

Within this population, conduct disorder and substance use disorders consistently emerge as the most prevalent diagnoses, frequently presenting in overlapping and highly comorbid patterns that complicate both clinical treatment and forensic decision-making (7, 8). Neurodevelopmental disorders, including attention-deficit/hyperactivity disorder, are also overrepresented and contribute to persistent difficulties in impulse control, emotional regulation, and behavioral adaptation (9, 10). At the same time, internalizing disorders such as depression, anxiety, and post-traumatic stress disorder are often under-recognized in forensic contexts, despite their substantial impact on affect regulation and risk behavior (11, 12). The presence of unrecognized or inadequately treated psychiatric conditions undermines the effectiveness of purely punitive responses and reinforces the necessity of integrated mental health approaches within juvenile justice systems (13).

Although the association between juvenile offending and psychiatric morbidity has been extensively investigated in Western Europe and North America, empirical data from Central and Eastern Europe remain comparatively limited. This lack of region-specific evidence constrains the development of context-sensitive prevention and intervention models, particularly in healthcare systems where forensic child and adolescent psychiatry services are still evolving (14, 15). Consequently, systematic investigation of the psychiatric profiles, comorbidity patterns, and hospitalization trajectories of forensically involved adolescents within inpatient psychiatric settings remains a critical unmet need in this geographical region.

The relationship between forensic involvement and mental health is increasingly conceptualized within a developmental psychopathology framework, which emphasizes the dynamic interaction between individual vulnerabilities and environmental risk factors across critical developmental stages (16, 17). Within this perspective, antisocial and high-risk behaviors are viewed not as isolated phenomena, but as outcomes of cumulative biopsychosocial processes involving neurodevelopmental deficits, adverse childhood experiences, family dysfunction, and social marginalization (18, 19).

Central to this conceptualization is the victim–offender overlap hypothesis, which posits that adolescents who engage in delinquent behavior are frequently exposed to earlier trauma, neglect, and chronic adversity (20, 21). Experiences of physical abuse, emotional maltreatment, sexual victimization, and sustained household instability significantly increase the risk of developing externalizing psychopathology, impaired stress regulation, and maladaptive coping strategies such as substance use and aggression (22, 23). Neurobiological studies further demonstrate that chronic early stress alters brain systems responsible for executive functioning, emotional regulation, and reward processing, thereby reinforcing vulnerability to impulsive and high-risk behaviors (24, 25).

In response to these insights, contemporary forensic psychiatry increasingly advocates for trauma-informed and developmentally sensitive approaches that prioritize therapeutic engagement and rehabilitation over exclusive reliance on punitive containment (13, 26). Such approaches emphasize the importance of early identification, comprehensive psychiatric assessment, and integrated intervention strategies that address both mental health needs and criminogenic risk factors within a coordinated framework.

Grounded in these developmental and theoretical perspectives, the present study examines the mental health characteristics of forensically involved adolescents receiving inpatient psychiatric care over a ten-year period in a national referral center in Hungary. By integrating standardized diagnostic assessment with epidemiological analysis, the study aims to provide region-specific insight into psychiatric morbidity, comorbidity patterns, and readmission dynamics within this high-risk population, thereby supporting more informed clinical and forensic decision-making in Central and Eastern Europe.

The primary objectives of the study were to: (1) determine the prevalence and pattern of psychiatric diagnoses and comorbidities among forensically involved adolescents requiring inpatient care; (2) examine demographic and clinical factors associated with hospitalization characteristics; and (3) explore readmission dynamics as an indirect indicator of illness severity and continuity of care. Based on existing international literature, we hypothesized that: (1) externalizing disorders, particularly conduct disorder and substance use disorders, would be highly prevalent; (2) psychiatric comorbidity would be widespread; (3) older adolescents would exhibit shorter time-to-readmission intervals compared to younger age groups; and (4) the clinical profiles of forensically involved adolescents would reflect greater severity than those typically observed in general inpatient child and adolescent psychiatric populations. Acute indications for admission to closed inpatient child and adolescent psychiatric care include psychotic or manic episodes, severe suicidal behavior or non-suicidal self-injury, and behavioral dysregulation posing immediate risk to the patient or others.

Through addressing these aims and hypotheses, this study seeks to contribute robust empirical evidence supporting the advancement of trauma-informed, developmentally sensitive, and interdisciplinary models of care within forensic child and adolescent psychiatry, bridging clinical practice and juvenile justice systems.

The aims of this study were to characterize the clinical and diagnostic features of adolescents admitted to inpatient psychiatric care and to explore readmission dynamics as an indirect indicator of clinical instability across age groups.

## Methods

### Study design and setting

This retrospective cohort study was conducted at the Department of Child and Adolescent Psychiatry of the Heim Pál National Institute of Pediatrics, the national tertiary referral center for inpatient child and adolescent mental health care in Hungary. Ethical approval was obtained from the Institutional Research Ethics Committee (IKEB; approval KUT-27/2021). All analyses were conducted on fully anonymized data in accordance with GDPR and the Declaration of Helsinki.

### Participants and case identification

Medical records for all adolescents admitted between 1 July 2009 and 30 June 2019 were screened using the hospital’s electronic medical record system (SanitasX™). A custom keyword-based search identified forensic involvement, defined as admission under police escort, judicial order, or documented reference to suspected or confirmed criminal behavior.

Of 6,438 admissions during the study period, 2,073 contained forensic references; after deduplication, 570 unique patients aged 10–19 years comprised the final cohort. The lower age limit (10 years) captured the developmental “upswing phase” preceding the 13–17-year peak of juvenile offending.

Age groups (10–12, 13–15, and 16–18 years) were selected to reflect distinct developmental stages that carry differential psychiatric and behavioral risk profiles in adolescence. These categories also align with known forensic-relevance thresholds, given that juvenile offending peaks between ages 13 and 17, making these bins developmentally and clinically meaningful for subgroup comparisons.

### Diagnostic instruments

Psychiatric diagnoses and symptom profiles were assessed using standardized and internationally validated instruments routinely applied in the clinical setting:

- Mini International Neuropsychiatric Interview for Children and Adolescents (MINI-Kid): A structured diagnostic interview based on DSM-IV/DSM-5 and ICD-10 criteria, covering a broad spectrum of psychiatric disorders, including externalizing, internalizing, substance-related, and neurodevelopmental conditions. (27).
- Child Behavior Checklist (CBCL): A parent-reported questionnaire assessing emotional and behavioral problems across internalizing and externalizing domains.
- Strengths and Difficulties Questionnaire (SDQ): A brief screening tool measuring emotional symptoms, conduct problems, hyperactivity, peer relationship difficulties, and prosocial behavior (28, 29).
- Attention was assessed using the **Piéron Attention Test**, a cancellation-based measure widely applied in clinical and neuropsychological assessment of attentional functioning (30, 31).

All instruments had been previously validated and culturally adapted for use in Hungarian child and adolescent clinical populations.

### Data processing and variables

Multiple hospital admissions belonging to the same individual were merged into unique patient profiles. Data cleaning and structural standardization were performed using Microsoft Excel, followed by statistical preparation and analysis in IBM SPSS Statistics. Extracted variables included age, sex, psychiatric diagnoses, number of admissions, length of hospitalization, and interval between admissions.

### Statistical analysis

Statistical analyses were conducted using IBM SPSS Statistics version 26.0 (IBM Corp., Armonk, NY, USA). Descriptive statistics were calculated for demographic and clinical variables, including frequencies, percentages, means, standard deviations, medians, and ranges.

Group comparisons were performed using:

- Chi-square tests (χ²) for categorical variables,
- Independent-samples t-tests or Mann–Whitney U tests for continuous variables, as appropriate based on distribution characteristics.

Readmission intervals were analyzed both as categorical time frames and as continuous time-to-event data. Kaplan–Meier survival analysis was applied to estimate time-to-readmission, and group differences were examined according to age and sex using log-rank tests. Statistical significance was defined as p < 0.05 for all analyses.

Age stratification (≤11.9, 12.0–13.9, and ≥14 years) was based on established developmental and forensic-risk thresholds. These boundaries reflect transitions in cognitive maturation, autonomy, and exposure to criminogenic environments, which are known to influence both relapse risk and psychiatric destabilization patterns in justice-involved adolescents.

## Results

### Sample characteristics

The final study cohort comprised 570 forensically involved adolescents admitted to inpatient psychiatric care between 2009 and 2019. The mean age was 15.3 years (SD ± 2.1), and the sample was predominantly male (72.1%), with females comprising 27.9%. Age distribution demonstrated a concentration in mid-to-late adolescence, with the highest admission rates observed in the 15–17-year age range.

Most patients (75.0%) were hospitalized once during the study period; however, 25.0% experienced two or more admissions, indicating a subgroup with recurrent psychiatric crises or incomplete clinical stabilization following discharge.

### Psychiatric diagnoses and comorbidity

Psychiatric morbidity was highly prevalent across the cohort. The most frequently diagnosed conditions were substance use disorders (78.4%) and conduct disorder (76.2%), followed by attention-deficit/hyperactivity disorder (17.6%), oppositional defiant disorder (14.9%), post-traumatic stress disorder (10.1%), and psychotic disorders (3.2%).

Comorbidity was common, with more than two-thirds of the cohort presenting with at least two co-occurring psychiatric diagnoses. The most frequent diagnostic combination was conduct disorder co-occurring with substance use disorder. Internalizing disorders were less frequently diagnosed but remained clinically relevant, particularly when occurring alongside externalizing symptom profiles.

Across the cohort, 1,142 diagnoses were recorded among 570 patients, corresponding to a mean of 2.00 diagnoses per patient.

### Hospitalization patterns

The mean length of inpatient stay did not differ substantially from that of the general adolescent psychiatric inpatient population treated at the same institution during the study period. Nevertheless, the pattern of repeated admissions suggested the presence of a clinically unstable subgroup characterized by chronic or relapsing psychopathology.

The interval between discharge and subsequent readmission showed considerable variability. Younger adolescents tended to demonstrate longer time intervals prior to rehospitalization, whereas older adolescents exhibited significantly shorter intervals, indicating increased vulnerability to rapid clinical destabilization with advancing age.

### Readmission dynamics and survival analysis

Kaplan–Meier survival analysis revealed a progressive decline in admission-free survival over time among patients with recurrent hospitalization. While no statistically significant differences were observed between males and females in time-to-readmission (log-rank test, p > 0.05), age emerged as a significant predictor of earlier rehospitalization. Adolescents aged 16 years and older demonstrated steeper survival curves, reflecting earlier and more frequent readmissions compared to younger age groups.

The early stepwise intervals (1.5, 13, and 75 days) reflect institutional reporting bins and structured follow-up checkpoints used in the inpatient unit, which determine how readmission events are grouped in the medical record system.

These findings indicate that increasing age is associated with a higher risk of rapid relapse requiring inpatient psychiatric care, highlighting an age-related escalation in clinical vulnerability within this population.

### Summary of key findings

Overall, the findings of this ten-year retrospective cohort study demonstrate that forensically involved adolescents requiring inpatient psychiatric care exhibit an exceptionally high burden of psychiatric disorders, predominantly involving externalizing and substance-related conditions. **Table 1** summarizes the prevalence of key psychiatric diagnoses within the cohort, highlighting particularly high rates of substance use disorder (78.4%) and conduct disorder (76.2%).

**Table 1:** Psychiatric diagnoses among forensically involved adolescents (N = 570).

Diagnosis	n	%
Substance use disorder (SUD)	447	78.4
Conduct disorder (CD)	434	76.2
Attention-deficit/hyperactivity disorder (ADHD)	100	17.6
Oppositional defiant disorder (ODD)	85	14.9
Post-traumatic stress disorder (PTSD)	58	10.1
Psychotic disorders (e.g., schizophrenia spectrum)	18	3.2

**Table 1** presents the prevalence and percentage distribution of major psychiatric diagnoses identified in the study cohort. Diagnostic categories include substance use disorder, conduct disorder, attention-deficit/hyperactivity disorder, oppositional defiant disorder, post-traumatic stress disorder, and psychotic disorders. Percentages refer to the full “researched” cohort of 570 adolescents aged 10–18 years treated at the HOGYI Child and Adolescent Psychiatry Unit between July 1, 2009 and June 30, 2019.

**Figure 1:**
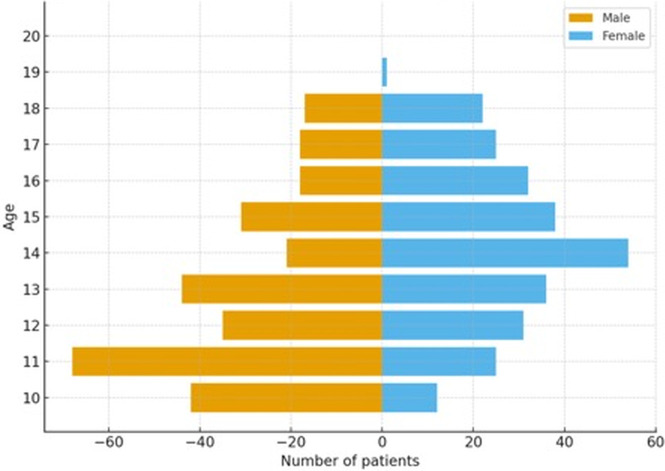
Age distribution in the forensically involved inpatient cohort (N = 570). **Figure 1** depicts the age distribution across three developmental age bands (10–12, 13–15, and 16–18 years) based on the complete study sample (M = 13.98 years, SD = 2.40). These groupings reflect clinically and forensically meaningful developmental thresholds used throughout the analyses.

**Figure 1** presents the age distribution of the full forensic inpatient cohort. Admission frequencies were highest in mid-to-late adolescence, consistent with known developmental risk windows for offending and psychiatric destabilization.

**Figure 2:**
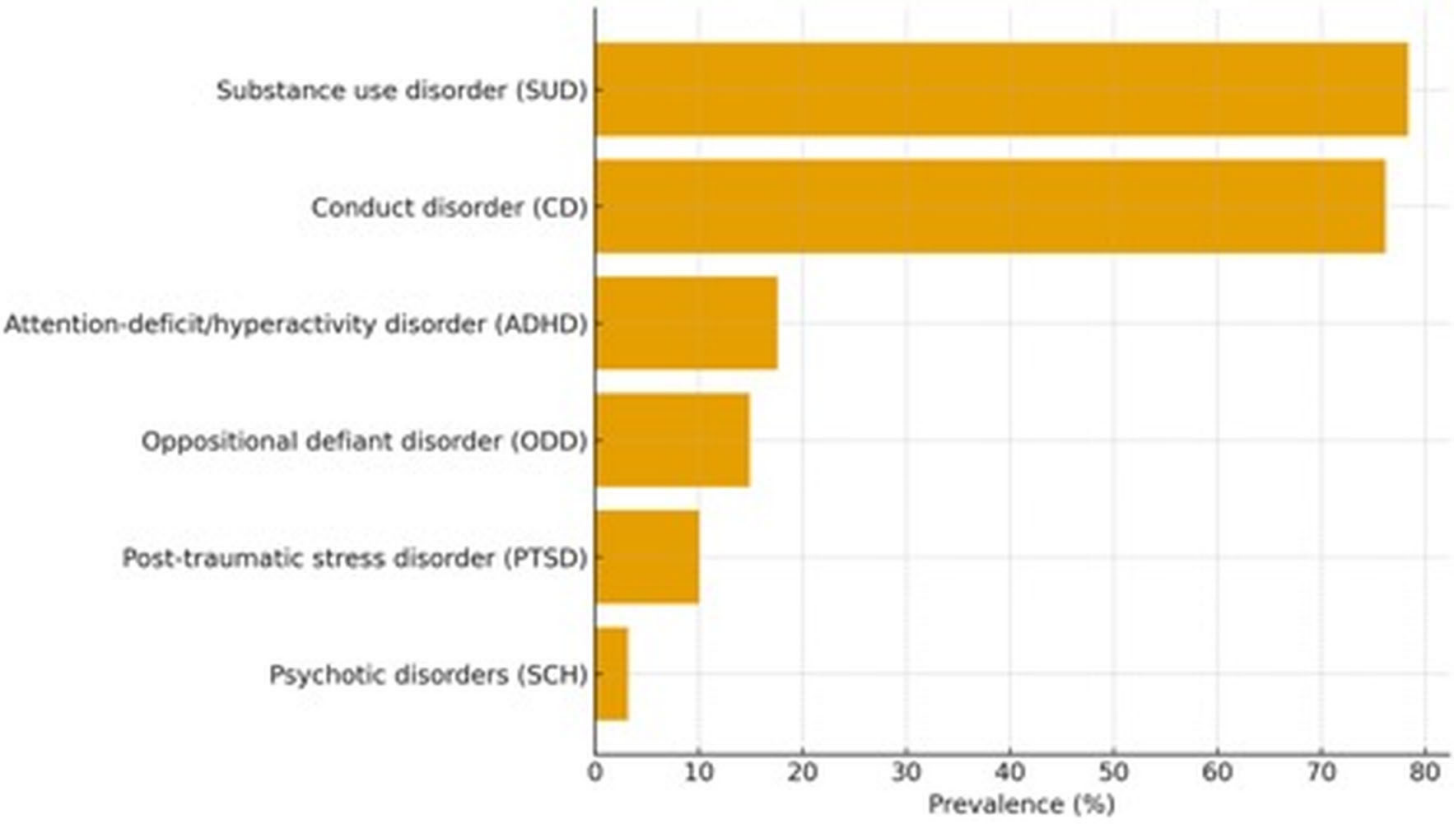
Diagnostic patterns in the forensically involved inpatient cohort (N = 570). **Figure 2** illustrates the prevalence of key psychiatric diagnoses as defined by DSM-IV/DSM-5 criteria over the ten-year study period. The bar chart displays conduct disorder (76.2%), substance use disorder (78.4%), attention-deficit/hyperactivity disorder (17.6%), oppositional defiant disorder (14.9%), post-traumatic stress disorder (10.1%), and psychotic disorders (3.2%).

Diagnostic patterns for major psychiatric categories are visualized in **Figure 2**. The distribution reinforces the dominant role of externalizing and substance-related psychopathology within this population.

**Figure 3:**
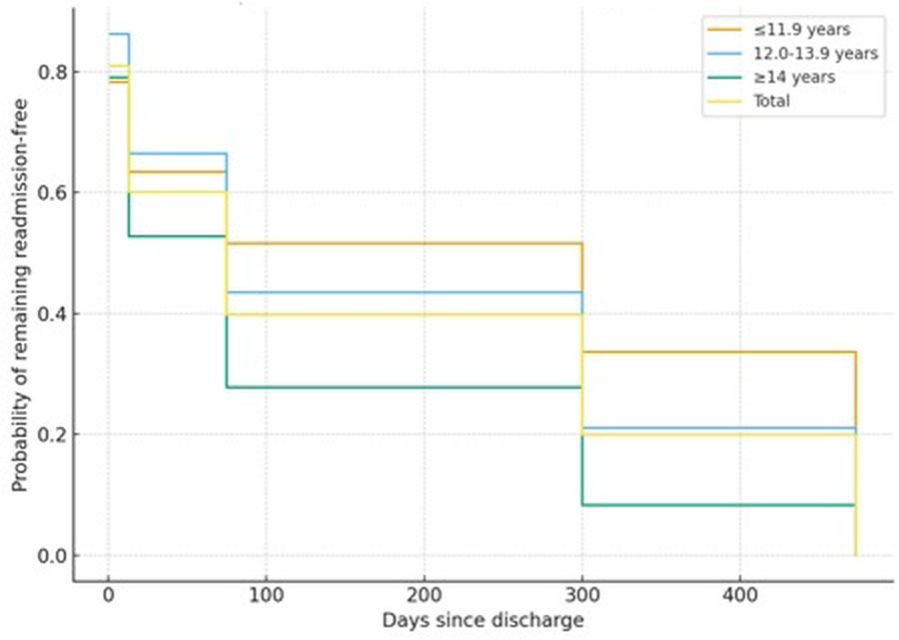
Kaplan–Meier analysis of time to readmission stratified by age group. **Figure 3** presents Kaplan–Meier survival curves showing the probability of remaining readmission-free following discharge across three age groups (≤11.9 years, 12.0–13.9 years, ≥14 years). Stepwise changes reflect institutional follow-up interval bins (1.5, 13, 75, 300, and 473 days). Older adolescents (≥14 years) exhibited a significantly steeper decline in survival probability. Statistical analysis confirmed an association between age group and time-to-readmission (Pearson χ² = 47.2, df = 8, p < 0.001). (Age group boundaries reflect analytical bins used in survival modeling and may differ slightly from descriptive age categories.).

Recurrent hospitalization affected one in four patients, and analyses revealed a clear age-related gradient in the timing of psychiatric relapse. Older adolescents demonstrated markedly shorter readmission intervals, reflecting increased clinical vulnerability and diminished stabilization following discharge. These patterns are visualized in **Figure 3**.

These results collectively underscore the high clinical complexity and instability characteristic of forensically involved adolescents requiring inpatient psychiatric care.

## Discussion

### Summary of principal findings

This study provides a comprehensive characterization of the psychiatric profiles and hospitalization trajectories of forensically involved adolescents treated in a national inpatient child and adolescent psychiatry unit over a ten-year period. The findings reveal a population marked by exceptionally high rates of psychiatric morbidity, substantial comorbidity, and clinically significant instability, as reflected in recurrent hospitalizations and age-related vulnerabilities in readmission patterns. Externalizing and substance-related disorders emerged as the dominant diagnostic categories, while internalizing and trauma-related conditions—though less frequently recorded—remained clinically consequential. Together, these patterns underscore the complex and multifaceted nature of psychiatric impairment in justice-involved youth. The diagnostic approach applied in this study aligns with established clinical assessment frameworks using standardized instruments that have been extensively validated in child and adolescent psychiatric populations, including high-risk and justice-involved youth samples (3, 5, 37–39).

### Psychiatric burden and diagnostic patterns

The prevalence of conduct disorder and substance use disorders in this cohort exceeded rates typically reported in both community populations and several international forensic samples, highlighting the severity of externalizing psychopathology among adolescents requiring inpatient care. The high comorbidity between these disorders aligns with prior research demonstrating their mutually reinforcing nature and their association with adverse long-term outcomes, including persistent antisocial behavior and increased risk of reoffending.

Neurodevelopmental conditions—including attention-deficit/hyperactivity disorder and oppositional defiant disorder—were also prominently represented. Their contribution to impulsivity, affective dysregulation, and functional impairment likely exacerbates both clinical presentation and forensic risk. Although internalizing disorders appeared less frequently in diagnostic summaries, the presence of post-traumatic stress disorder in more than one-tenth of the sample underscores the critical role of trauma exposure as a transdiagnostic factor shaping behavioral and emotional functioning in this population. This pattern supports the broader empirical consensus that disruptive or aggressive behavior in forensic contexts may obscure underlying emotional distress, maladaptive coping strategies, or trauma-related symptoms.

### Developmental interpretation

These findings may reflect cumulative developmental vulnerability, consistent with developmental psychopathology models emphasizing the interaction of early risk exposure, symptom continuity, and age-specific manifestations of psychopathology across adolescence (16, 32). In this perspective, externalizing behaviors, substance misuse, and repeated psychiatric crises are not viewed as isolated phenomena, but as manifestations of disrupted developmental trajectories shaped by early and persistent risk factors.

Importantly, the present results do not imply a direct or deterministic pathway toward formal justice-system involvement. Rather, the clinical profiles observed in this cohort reflect severe developmental vulnerability within a high-risk inpatient population, may encounter coercive or crisis-driven interventions—such as emergency containment or police involvement—within the healthcare system rather than through judicial processes. The absence of justice-system trajectory data necessitates caution in interpreting these patterns, and the current findings should be understood as indicators of psychiatric risk rather than markers of legal outcome.

Consistent with prior research, the high prevalence of conduct disorder and substance use disorders suggests that early-onset behavioral dysregulation frequently co-occurs with maladaptive coping strategies and impaired impulse control. Neurodevelopmental conditions, including attention-deficit/hyperactivity disorder, may further amplify vulnerability by compromising executive functioning and emotional regulation during critical developmental periods. At the same time, the presence of trauma-related disorders highlights the role of adverse childhood experiences as transdiagnostic contributors to clinical instability. Trauma exposure and cumulative adversity are likely contributors to the observed clinical severity, consistent with evidence demonstrating that adverse childhood experiences exert dose–response effects on mental health vulnerability and symptom complexity across development (33, 34).

Repeated hospitalization may indicate clinical instability, consistent with evidence that acute inpatient admission and subsequent readmission in child and adolescent psychiatry are associated with greater symptom severity, complexity, and ongoing risk requiring intensive clinical management (35, 36).

Within this framework, acute psychiatric hospitalization emerges as a sentinel event, reflecting the convergence of longstanding developmental risk, psychosocial adversity, and escalating symptom severity. Rather than signaling isolated behavioral crises, repeated admissions may represent the clinical expression of entrenched vulnerability that intensifies with age, particularly in the context of diminishing external supports and increasing autonomy during late adolescence.

### Hospitalization and readmission dynamics

The pattern of hospitalization further highlights the clinical instability of this population. Although most adolescents were admitted only once, a significant proportion required multiple admissions, indicating persistent or recurrent psychiatric crises. The survival analysis revealed that older adolescents exhibited significantly shorter time-to-readmission intervals compared to younger peers, suggesting an age-related intensification of clinical vulnerability. Several mechanisms may account for this pattern, including prolonged exposure to adverse environments, escalation of substance use, entrenched behavioral patterns, and diminishing protective factors such as family supervision or school engagement. These findings point to the need for age-specific approaches in both inpatient management and discharge planning.

### Clinical implications

The exceptionally high burden of psychiatric morbidity and the observed patterns of recurrent hospitalization underscore the need for integrated, trauma-informed, and developmentally sensitive psychiatric care for high-risk adolescents requiring acute inpatient treatment. These findings highlight the limitations of crisis-driven or containment-focused responses that address immediate behavioral risk without adequately targeting underlying psychiatric and developmental needs.

From a clinical perspective, early identification of externalizing psychopathology, substance use, and trauma-related symptoms at the point of inpatient admission is critical. Comprehensive diagnostic assessment should be coupled with individualized treatment planning that extends beyond symptom stabilization to include long-term risk management, psychosocial support, and continuity of care following discharge. The strong association between increasing age and earlier readmission suggests that older adolescents represent a particularly vulnerable subgroup requiring intensified transitional planning and post-discharge monitoring.

Although some adolescents in this cohort were admitted following police or judicial contact, their treatment occurred entirely within the healthcare system. This underscores the importance of strengthening coordination between mental health services and external agencies, while maintaining a clear distinction between clinical care and judicial responsibility. Collaborative frameworks that facilitate information sharing, continuity of treatment, and access to community-based supports may reduce repeated crises and rehospitalization.

Overall, the findings support a shift toward proactive, rehabilitative models of care that prioritize psychiatric stabilization, trauma-sensitive intervention, and developmental support. By addressing the underlying drivers of clinical instability, such approaches may improve long-term outcomes for high-risk adolescents and reduce the likelihood of recurrent inpatient admissions.

### Strengths and limitations

The strengths of this study include its large clinical sample, decade-long observation period, and reliance on standardized diagnostic instruments within a national tertiary inpatient setting. These features enhance both the reliability and the clinical relevance of the findings. However, several limitations should be considered. The retrospective design precludes causal inference, and the exclusive focus on hospitalized adolescents limits generalizability to community or outpatient forensic populations. Diagnostic patterns may also be influenced by documentation practices and the prioritization of externalizing symptoms during acute admissions. Important social and environmental variables, including family structure, socioeconomic status, foster care placement, and involvement with child protection services, were not systematically available in the retrospective clinical records and could therefore not be included in the analyses. Additionally, the absence of long-term functional outcomes beyond readmission prevents assessment of broader trajectories of recovery or reoffending.

### Future directions

Future research should adopt prospective longitudinal designs to elucidate developmental trajectories, identify modifiable risk factors, and distinguish between persistent and desisting patterns of offending. Comparative analyses involving both forensic and non-forensic inpatient populations may clarify diagnostic features unique to justice-involved adolescents. Finally, intervention studies assessing the effectiveness of trauma-informed, developmentally tailored treatment approaches could provide essential evidence to guide best practices in forensic child and adolescent psychiatry.

## Conclusion

This study contributes to the existing literature by providing region-specific data from Central and Eastern Europe on adolescents requiring acute inpatient psychiatric care. By focusing on a high-risk inpatient population and quantifying diagnostic burden and readmission patterns, the findings highlight the clinical complexity and instability characteristic of this group. These results underscore the importance of developmentally sensitive, trauma-informed inpatient and post-discharge care pathways for adolescents at the most severe end of the psychiatric spectrum.

Overall, this study demonstrates that forensically involved adolescents requiring inpatient psychiatric care constitute a clinically severe and developmentally vulnerable population. The convergence of externalizing psychopathology, substance misuse, trauma exposure, and neurodevelopmental impairments reflects a multidimensional and deeply entrenched pattern of risk. These findings underscore the pressing need for integrated models of care that prioritize early detection, comprehensive assessment, and sustained post-discharge support. Strengthening continuity of care and bridging clinical and juvenile justice services represent critical steps toward improving psychiatric stability and reducing repeated system involvement for this high-risk group.

## Data Availability

The datasets presented in this article are not readily available because The dataset contains sensitive clinical information from minors and is subject to institutional, ethical, and legal restrictions. For this reason, the raw data cannot be shared. Requests to access the datasets should be directed to Gyula Sófi, drsofigyula@gmail.com.
